# Changes in the Electrical Characteristics of Perovskite Solar Cells with Aging Time

**DOI:** 10.3390/molecules25102299

**Published:** 2020-05-14

**Authors:** Apurba Mahapatra, Nishi Parikh, Pawan Kumar, Manoj Kumar, Daniel Prochowicz, Abul Kalam, Mohammad Mahdi Tavakoli, Pankaj Yadav

**Affiliations:** 1 Department of Physics & Astronomy, National Institute of Technology, Rourkela 769008, India; mahapatraapurba@gmail.com (A.M.); pawankumar@nitrkl.ac.in (P.K.); 2 Department of Science, School of Technology, Pandit Deendayal Petroleum University, Gandhinagar 382 007, India; nishiparikh97@gmail.com (N.P.); Manoj.kspv@gmail.com (M.K.); 3 Institute of Physical Chemistry, Polish Academy of Sciences, 01-224 Warsaw, Poland; 4 Department of Chemistry, Faculty of Science, King Khalid University, P.O. Box 9004, Abha 61413, Saudi Arabia; abul_k33@yahoo.com; 5 Department of Electrical Engineering and Computer Science, Massachusetts Institute of Technology, Cambridge, MA 02139, USA; mtavakol@mit.edu; 6 Department of Solar Energy, School of Technology, Pandit Deendayal Petroleum University, Gandhinagar 382 007, India

**Keywords:** impedance spectroscopy, perovskite solar cells, recombination

## Abstract

The last decade has witnessed the impressive progress of perovskite solar cells (PSCs), with power conversion efficiency exceeding 25%. Nevertheless, the unsatisfactory device stability and current–voltage hysteresis normally observed with most PSCs under operational conditions are bottlenecks that hamper their further commercialization. Understanding the electrical characteristics of the device during the aging process is important for the design and development of effective strategies for the fabrication of stable PSCs. Herein, electrochemical impedance spectroscopical (IS) analyses are used to study the time-dependent electrical characteristics of PSC. We demonstrate that both the dark and light ideality factors are sensitive to aging time, indicating the dominant existence of trap-assisted recombination in the investigated device. By analyzing the capacitance versus frequency responses, we show that the low-frequency capacitance increases with increasing aging time due to the accumulation of charges or ions at the interfaces. These results are correlated with the observed hysteresis during the current–voltage measurement and provide an in-depth understanding of the degradation mechanism of PSCs with aging time.

## 1. Introduction

Perovskite solar cells (PSCs) have become the front runner in emerging thin-film solar cells, with power conversion efficiency (PCE) exceeding 25% owing to their low-cost solution processing and exciting optoelectronic features such as their high absorption coefficient, tunable stability, and superior carrier transportation properties [1,2,3,4,5,6,7,8]. Nevertheless, the key issue that hampers the commercialization of PSCs is device instability under light, moisture, and high-temperature exposure [9,10]. There are two types of instability for PSCs. One is the intrinsic chemical instability of the perovskite absorber layer, and the other is associated to the electronic properties of the device [10]. The latter includes ion migration and inefficient charge transport, which lead to the hysteresis phenomenon and light soaking under operational conditions [11].

Apart from the device architectures and perovskite formulations, the performance of PSCs highly depends on the morphology and quality of each compositional layer [12,13,14]. For instance, the large crystallite size of the perovskite absorber reduces the grain boundaries and trap states, leading to a decrease in non-radiative recombination and improved PCE [15,16,17]. Furthermore, in-depth investigations of the bulk and interface parameters using capacitance–voltage and frequency-dependent capacitance studies suggest that ion migration changes the inherent electric field and decreases the charge accumulation at the interfaces [18,19]. For example, Zhao et. al. demonstrated that upon light illumination, the bulk defects within the perovskite layer are positively charged and are neutralized by the photogenerated electrons, and that this process affects the open-circuit voltage (V_OC_) and fill factor (FF) of the device [18]. Moreover, the short-circuit current density (J_SC_) decreases in the light soaking test as the bulk electrical polarization is decreased within the perovskite film. On the other hand, Deng et al. showed that the performance of PSCs decreases due to the negative ions drifting to the spiro-OMeTAD/perovskite interface in a prolonged light soaking test [20]. In another study, the loss in performance was attributed to light-activated trap states [21]. Tress et al. reported that accumulation of cation vacancies at the electrode induced reversible performance losses [22]. To date, many mechanisms have been proposed to understand the phenomenon of light soaking by considering different device architectures, preparation methods, and properties of hole and electron extracting layers [23,24,25,26]. However, a clear explanation is still lacking and thus further studies on this topic are required to elucidate more in-depth conclusions.

In this work, we employed electrochemical impedance spectroscopical (IS) analyses for studying the PSC aging process in ambient conditions. The current–voltage (J–V) characteristics were measured to monitor the evolution of the photovoltaic parameters of the device over time. We found that the ideality factors calculated from either the dark and light current–voltage curves increased over aging time. The capacitance–frequency analysis depicted that the low-frequency capacitance increased with increasing aging time. The increase in capacitance at low frequencies resulted from the accumulation of charges or ions at the interfaces, which was well correlated with the observed hysteresis in the current–voltage characteristics of PSCs.

## 2. Results

A prototypical PSC with an architecture consisting of fluorine-doped tin oxide (FTO)/compact TiO_2_/mesoporous TiO_2_/MAPbI_3_/spiroOMeTAD/Au was prepared to study time-dependent device performance and stability (for details on the device fabrication procedure see the Experimental Section). The thicknesses of the perovskite absorber and hole transporting layers were around 250 nm and 150 nm, respectively (Figure S1). The current–voltage (J–V) characteristics of the device were measured under air mass 1.5 global (AM 1.5 G) irradiance using the Bio-Logic galvanostat in the dark and with different light intensities (reverse scanning at room temperature). The electrical properties of the freshly prepared device (further denoted as a reference) were measured as a function of time after 24 h and 94 h with the help of impedance spectroscopy techniques. Before J–V measurement, the device was stored in the ambient relative humidity (RH) of 20% in the dark. In our previous work, we demonstrated that the performance of PSCs could be also hampered by the applied electrical potential [27]. Thus, a minimum duration bias followed by a long recovery time was applied to avoid the bias effect.

**Figure 1:**
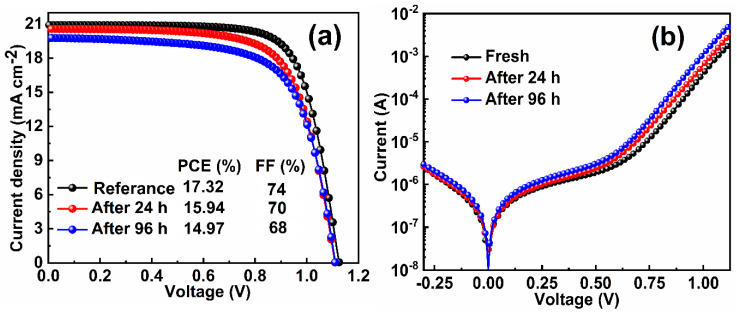

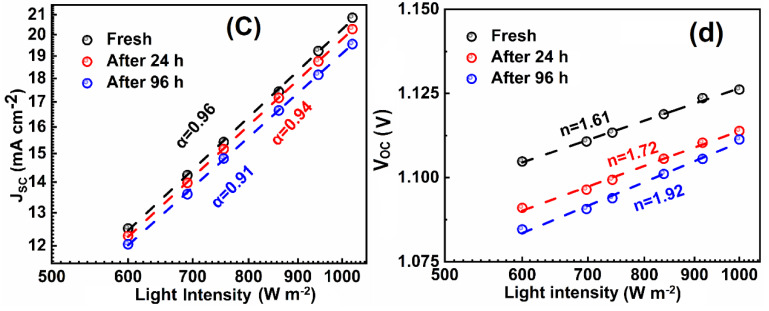
The current–voltage (J–V) measurements of the perovskite solar cell (PSC) as a function of time under (**a**) AM 1.5 G light intensity and reverse scanning. (**b**) Dark J–V curves of the corresponding devices at room temperature. Light intensity dependence of (**c**) short-circuit current density (J_SC_) and (**d**) V_OC_ under reverse scanning in the investigated devices.

The J–V curves of the device measured over time and the extracted photovoltaic parameters are shown in Figure 1a and Table 1, respectively. The reference device exhibited a J_SC_ of 20.91 mA cm^−2^, V_OC_ of 1.12 V, FF of 74%, and PCE of 17.3%. In turn, the 96-h aged device showed reduced values of J_SC_, V_OC_, FF, and PCE, which were 19.7 mA cm^−2^, 1.11 V, 68%, and 14.97%, respectively. It was observed that the reduction in PCE was mainly related to the lowering of FF and J_SC_.

To obtain more insight into the reduction of these parameters, a dark J–V measurement of the PSC at room temperature (300 K) was performed and is shown in Figure 1b. A lower magnitude of the net current obtained for the reference device as compared to the aged devices signified that upon aging, a higher charge recombination rate took place in the device. The ideality factors (*n*) of the PSCs were calculated from the semi-log J–V plots in the dark using Equation (1):

(1) $$n=1/\left(\frac{q}{kT}\;\frac{\partial \;J}{\partial \ln V}\right)$$

where k, T, q, J, and n are Boltzmann constant, temperature, charge of the electron, current density, and ideality factor, respectively (Figure 1b) [22,28]. The ideality factor values for the reference, 24-h, and 94-h aged PSCs were calculated to be 2.69, 2.73, and 2.84, respectively. The increase in the value of the ideality factor over time suggested the enhancement of trap states upon aging [28].

Illumination-dependent photovoltaic parameters were measured to further study the charge extraction and recombination in the investigated device. The normalized J–V characterizations of the reference and the devices after 24-h and 96-h aging times under various illumination intensities are shown in Figure S2. The variation in J_SC_ with light illumination is shown in Figure 1c. By using the power-law dependency of J_SC_ on light intensity, a slope value in the range of α = 0.96 to 0.91 was obtained. A decrease in the values of α indicates that the charge extraction is reduced with aging time [29]. The extracted values of the V_OC_ in PSCs as a function of light intensity measurements are shown in Figure 1d. From the obtained plot, the slope value was calculated by using Equation (2):

(2) $$n=\frac{q}{\mathrm{kT}}\;\frac{\partial {\;V}_{\mathrm{OC}}}{\partial \ln I}$$

where k, T, q, I, and n are the Boltzmann constant, temperature, charge of the electron, intensity of incident light, and ideality factor, respectively [30]. The ideality factor value was found to be 1.61, 1.72, and 1.92 for the reference and the devices aged for 24 and 96 h, respectively. In general, the dominance of trap-assisted [31] and bimolecular recombination [32] can be categorized by analyzing the value of ideality factor (*n*). The unity value of *n* proposes the dominance of recombination at the surface of the perovskite film and 1 < *n* < 2 suggests the presence of trap-assisted recombination in the PSC [22]. The higher ideality factor value of the devices aged for 24 and 96 h indicates the dominant existence of trap−assisted recombination.

**Figure 2:**
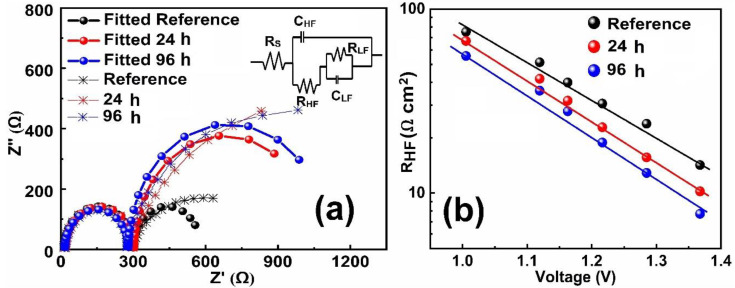
(**a**) A commonly used electrical equivalent circuit for impedance spectroscopy (IS) fitting of the PSCs and the IS spectra measured at built-in potential (V_bi_) under AM 1.5 G light illumination. (**b**) High-frequency resistance (R_HF_) vs. voltage under AM 1.5 G light illumination over time.

To shed more light on the origin of higher ideality factor observed for aged PSCs, impedance spectroscopy (IS) as a function of the applied bias was recorded under AM 1.5 G light illumination. The IS spectra was measured at frequencies ranging from 0.1 Hz to 1 MHz with 20 mV perturbation at room temperature (300 K). Figure S3 shows the bias-dependent IS spectra of the cells measured under AM 1.5 G light illumination. The commonly used electrical equivalent circuit for IS spectra fitting of the PSCs is shown in Figure 2a. Figure 2a shows the IS spectra measured at built-in potential (V_bi_) under AM 1.5 G light illumination. The description and physical significance of the used electrical equivalent circuit has been discussed in detail in several works, including our own [27,33,34]. Briefly, the R_S_ defines the series resistance, due to the electrical ohmic contacts. The high-frequency capacitance (C_HF_) and resistance (R_HF_) are due to the geometrical capacitance and recombination resistance. The low-frequency capacitance C_LF_ is attributed to the ionic response of accumulation or ion motion. The origin of low-frequency resistance R_LF_ is still a controversial topic in the literature and can be attributed to the surface resistance, accumulation resistance, and recombination resistance [35]. By using the electrical equivalent circuit, the value of R_S_ was determined to be 13, 15, and 18 Ω cm^2^ for the reference and devices with 24- and 94-h aging times, respectively. The estimated values of R_S_ from IS measurements were 13, 16, and 19 Ω cm^2^ for the reference and devices with 24- and 94-h aging times, respectively. Those values were comparable to the R_S_ values obtained from a single diode model. The higher series resistance suggests that the barrier for the transport of photogenerated charge carriers is increased in aged devices. Therefore, photogenerated charge extraction is reduced with time, leading to decreased FF [27]. As mentioned in the literature, the migrated ions from the perovskite layer can react with the selective contacts and restrict charge transport [29,36]. The fitted values of R_HF_ as a function of applied bias for all the PSCs are shown in Figure 2b. In general, a higher value of R_HF_ in PSCs defines the lower recombination rate. It was found that the reference device had the highest value of R_HF_ in the probed bias range, indicating a lower recombination. This result further confirmed the findings of the dark J–V and V_OC_ vs. illumination measurements. In the literature, it was shown that the recombination in PSCs could be also accelerated by the ions migration within the absorber layer [27]. To further confirm this fact, J−V hysteresis and capacitance−frequency (C–F) measurements were performed.

**Figure 3:**
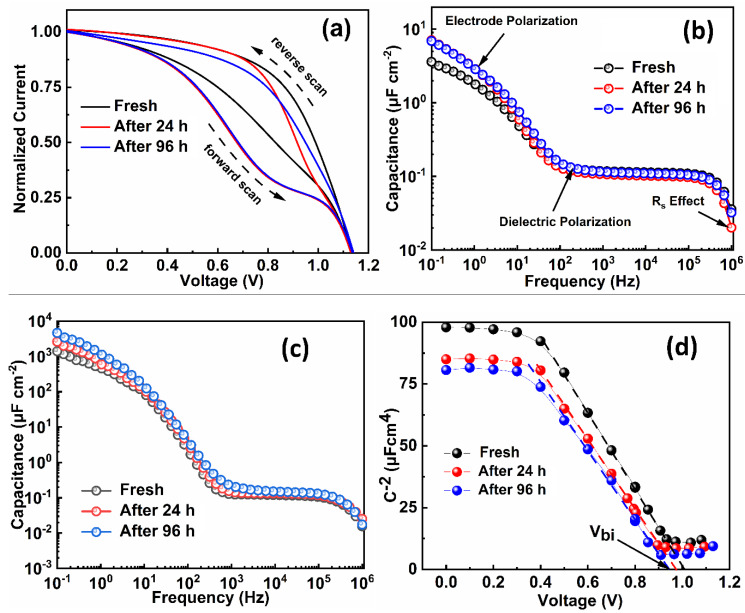
(**a**) Normalized J−V hysteresis characteristics of the device under AM 1.5 G light illumination as a function of time. Capacitance–frequency response of the device in a frequency range of 100 mHz to 1 MHz with time under (**b**) dark and (**c**) light. (**d**) Mott–Schottky plots of the investigated devices at 1 kHz.

Figure 3a shows the normalized J–V hysteresis of the PSCs measured under AM 1.5 G light illumination as a function of aging time. As reported elsewhere [37], the hysteresis loop between the forward and reverse scanning was related to the ion migration or accumulation phenomena. The hysteresis effect of the PSCs increased with time and maximized after 96 h, suggesting that the ion migration and charge accumulation in the device enhanced over time. The hysteresis behavior observed in the device was also correlated with the magnitude of low-frequency capacitance [37]. The C–F spectra of the device at zero bias in dark and under 1.5 G light illumination are shown in Figure 3b,c, respectively. In the obtained C–F plots, three distinct regions are clearly visible. The capacitance at low frequency was related to the electrode polarization, ions, and charge accumulation [35,38]. As shown in Figure 3b, the low−frequency capacitance was enhanced with the aging time, suggesting an increase in ion accumulation [35]. Jacobs et al. reported that the low-frequency capacitance under illumination was directly related to the recombination rate [39]. The higher value of low−frequency capacitance in aged devices is attributed to the higher accumulation of charges or ions at the interfaces and could be responsible for the observed high ideality factor in these devices. In the high-frequency range (<10^4^ Hz), the series resistance (R_S_) of the device reduced these capacitive responses. The higher value capacitance at 1 MHz in the aged devices clearly suggested that the series resistance increased with the aging time. This further confirmed that the electrical contact between the electrodes and cell deteriorate with aging. In turn, the capacitance values in the frequency range of 10^2^−10^5^ Hz defined as depletion layer capacitance plateau remained unaffected in all devices.

To understand the origin of the degraded V_OC_ of the aging device, the Mott–Schottky study was performed at a constant 1 kHz frequency, and built-in potential (V_bi_) could be determined by fitting the linear part of the C^−2^-V curves using Equation (3):

(3) $$\frac{1}{C^{-2}}=\frac{2}{A^{2}Nq\varepsilon \varepsilon_{0}}\left(V_{bi}-V\right)$$

where C, V, A, N, q, ε, and $\varepsilon_{0}$ are the measured capacitance, applied bias, active area, the concentration of donor-dopant, elementary charge, relative permittivity, and permittivity of free space, respectively [40]. The Mott–Schottky (M–S) plots of the reference and aged devices are shown in Figure 3d. As seen, the linear fit is done to obtain the built-in potential (V_bi_) of the device [38,41]. The calculated V_bi_ values from the intercepts were 1.02, 0.97, and 0.93 V for the reference and 24-h and 94-h aged devices, respectively. The V_bi_ is necessary for efficient charge extraction and avoids recombination [42]. A high value of V_bi_ allows the solar cell to reach a high V_OC_. The value of V_bi_ decreased for the aged devices, suggesting that the recombination increased with time, resulting in a drop in the V_OC_ of the cell. Moreover, the slope of the Mott–Schottky (M–S) plot provides the value of doping density in the PSCs [43]. In the present case, almost same values of the slope were observed, suggesting that the aging time had no significant impact on the doping density.

## 3. Materials and Methods

### 3.1. Solar Cell Device Fabrication

Fluorine-doped tin oxide (FTO)-coated glass substrates were cleaned by ultrasonic treatment in 2% Hellmanex water solution for 30 min and rinsed with deionized water and ethanol, and were then UV ozone-treated for 15 min before fabrication. The compact TiO_2_ layer was deposited by spray pyrolysis using 9 mL of ethanol solution containing 0.6 mL of titanium diiso-propoxide bis(acetylacetonate) solution (75% in 2-propanol, Sigma-Aldrich) and 0.4 mL acetylacetone at 450 °C in air [44]. On top of this layer, a 150-nm-thick mesoporous titanium dioxide layer was prepared by spin-coating 30-nm nanoparticles (Dyesol 30NRD, Dyesol) diluted in ethanol (1:6 wt/wt) at 5000 rpm for 10 s. The films were then gradually heated to 500 °C and sintered at that temperature for 1.5 h under oxygen atmosphere. The perovskite precursor solution (1.4 M in DMSO) was prepared from PbI_2_ (0.64 g) by mixing with methylammonium iodide (MAI, 0.22 g) in a molar ratio of 1:1 by vigorous stirring at 60 °C. The perovskite solutions were spin-coated in a two-step program at 1000 and 6000 rpm for 10 and 20 s, respectively. During the second step, 100 μL of chlorobenzene was poured on the spinning substrate 10 s prior to the end of the program [45]. The substrates were then annealed at 100 °C for 30 min in a dry box. The hole-transporting material (HTM) solution was prepared by dissolving 74 mg spiro-MeOTAD in 1 mL of chlorobenzene and additionally mixing it with 17.5 µL of lithium bis(trifluoromethylsulphonyl)imide (stock solution Li-TFSI 520 mg·mL^−1^ in acetonitrile), 28.8 µL tert-butylpyridine, and 29 μL of tris(2-(1H-pyrazol-1-yl)-4-tert-butylpyridine)cobalt(III) bis(trifluoromethylsulphonyl) imide (stock solution FK 209, 300 mg·mL^−1^ in acetonitrile). Subsequently, the HTM was deposited on top of the perovskite layer by spin coating at 4000 rpm for 20 s. Finally, 80 nm of gold top electrode was thermally evaporated under high vacuum. The active area of the devices was approximately 0.16 cm^2^.

### 3.2. Device Characterization

The J–V characteristics of the devices were measured under 100 mW/cm^2^ conditions (AM 1.5 G) using a 450 W Xenon lamp (Oriel) as a light source, equipped with a Schott K113 Tempax sunlight filter (Praezisions Glas & Optik GmbH, Iserlohn, Germany) to match the emission spectra to the AM 1.5 G standard in the region of 350–750 nm. The current–voltage characteristics of the devices were obtained by applying external potential bias to the cell while recording the generated photo-current using a Keithley (Model 2400, Cleveland, OH, USA) digital source meter. The J–V curves of all devices were measured by masking the active area with a metal mask of area 0.16 cm^2^. AC measurements were performed using a potentiostat Biologic SP300 equipped with a frequency response analyzer. We measured the IS measurements in the frequency range from 100 mHz to 1 MHz under AM 1.5 G light illumination conditions in the bias range from V_bi_ (0.85 V) to V_OC_ (1.13 V) under fresh conditions and then in dark conditions for 24 h, with further measurement and subsequent maintenance for 72 h.

## 4. Conclusions

In conclusion, we showed that the photovoltaic and electrical characteristics of the PSC changed during aging time. The observed reduction in PCE of the investigated device after 96-h exposure to ambient conditions was mainly related to the lowering of FF and J_SC_. The time-dependent measurements of the dark and light ideality factors revealed the dominant existence of trap-assisted recombination. Moreover, the analysis of the capacitance–frequency responses demonstrated that the low-frequency capacitance increased with increasing the aging time, which was attributed to the accumulation of charges or ions at the interfaces. The change in the capacitance clearly correlated with the observed hysteresis during the J–V measurements. The present study provides an in-depth analysis on the change in the electrical characteristics of PSCs with aging time, which will be crucial for designing the stable solar cells.

## Figures and Tables

**Table 1:** Photovoltaic parameters of the investigated PSC aged over time (reverse scanning). FF: fill factor; PCE: power conversion efficiency.

Index	V_OC_ (V)	J_SC_ (mAcm^−2^)	FF (%)	PCE (%)	Ideality Factor (*n*) (Dark)	Ideality Factor (*n*) (Light)
0 min	1.128	20.91	74	17.32	2.69	1.64
24 h	1.110	20.57	70	15.94	2.73	1.72
96 h	1.111	19.78	68	14.97	2.84	1.92

## Supplementary Materials

The following are available online. Figure S1: The normalized J–V characterizations of the investigated devices under different levels of illumination. Figure S2: IS spectra of the investigated devices measured under AM 1.5 G light illumination as a function of bias from built-in potential (V_bi_) to V_OC_.
