# Supplementary material for: Changes in the Electrical Characteristics of Perovskite Solar Cells with Aging Time
Source: Molecules. 2020 May 14;25(10):2299. doi: 10.3390/molecules25102299 (PMC7288190; doi:10.3390/molecules25102299)
Supplement: Supplementary file 1 [file molecules-25-02299-s001.pdf]

# Electronic Supplementary Information

## Change in the Electrical Charactertics of Perovskite Solar Cells with Aging Time

*Apurba Mahapatra,[1] Nishi Parikh,[2] Pawan Kumar,[1] Manoj Kumar,[2] Daniel Prochowicz,[3,]* Abul Kalam,[4] Mohammad Mahdi Tavakoli [5] and Pankaj Yadav [6,]**

[1] Department of Physics & Astronomy, National Institute of Technology, Rourkela, 769008, India

[2] Department of Science, School of Technology, Pandit Deendayal Petroleum University, Gandhinagar-382 007, Gujarat, India

[3] Institute of Physical Chemistry, Polish Academy of Sciences, Kasprzaka 44/52, 01-224 Warsaw, Poland

[4] Department of Chemistry, Faculty of Science, King Khalid University, Abha 61413, P.O. Box 9004, Saudi Arabia

[5] Department of Electrical Engineering and Computer Science, Massachusetts Institute of Technology, Cambridge, MA 02139, USA

[6] Department of Solar Energy, School of Technology, Pandit Deendayal Petroleum University, Gandhinagar-382 007, Gujarat, India.

**E-mail:** dprochowicz@ichf.edu.pl; Pankajphd11@gmail.com

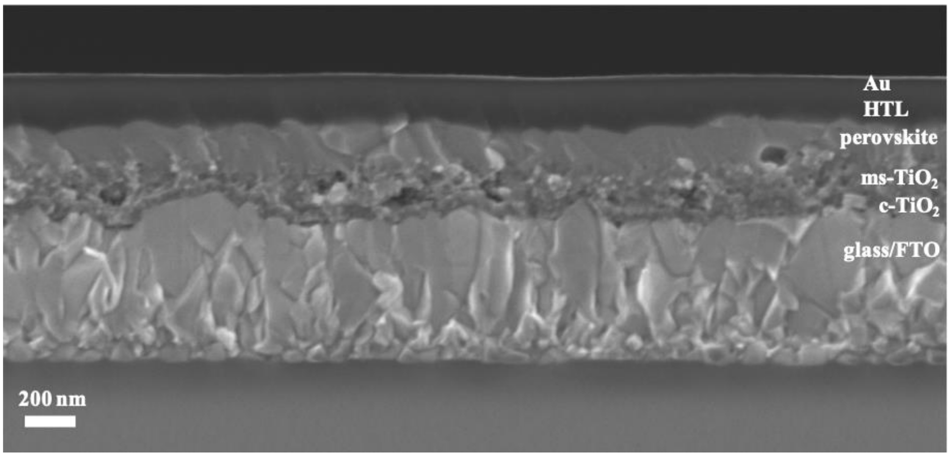


**Figure S1.** SEM cross section image of the investigated device.

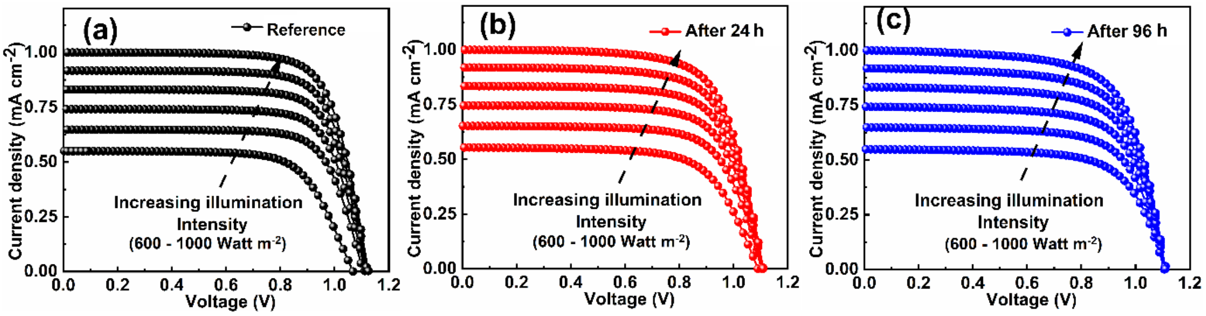


**Figure S2.** The normalized J-V characterizations of the (a) fresh, (b) 24 h and (c) 96 h aged device under different illumination.

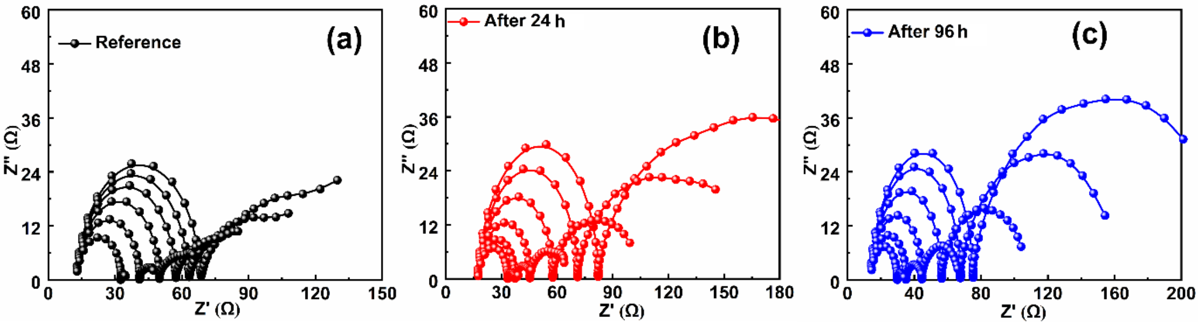


**Figure S3.** IS spectra of the (a) fresh, (b) 24 h and (c) 96 h aged device measured under AM 1.5G light illumination as a function of bias from 0.85-1.13V.
